# Publisher Correction: TGFβ signaling mediates microglial resilience to spatiotemporally restricted myelin degeneration

**DOI:** 10.1038/s41593-026-02361-6

**Published:** 2026-06-15

**Authors:** Keying Zhu, Yun Liu, Jin-Hong Min, Vijay Joshua, Jianing Lin, Yue Li, Judith C. Kreutzmann, Yuxi Guo, Wenlong Xia, Elyas Mohammadi, Melanie Pieber, Valerie Suerth, Yiming Xia, Zaneta Andrusivova, Jean-Philippe Hugnot, Shigeaki Kanatani, Per Uhlén, Joakim Lundeberg, Xiaofei Li, Stephen P. J. Fancy, Heela Sarlus, Robert A. Harris, Harald Lund

**Affiliations:** 1 Applied Immunology and Immunotherapy, Department of Clinical Neuroscience, Karolinska Institutet, Center for Molecular Medicine, Karolinska University Hospital, Stockholm, Sweden; 2 Department of Neurology, Division of Neuroimmunology and Glial Biology, University of California San Francisco, San Francisco, CA USA; 3 State Key Laboratory of Medical Neurobiology and MOE Frontiers Center for Brain Sciences, Institutes of Brain Science, Fudan University, Shanghai, China; 4 MOE Medical Basic Research Innovation Center for Gut Microbiota and Chronic Disease, Wuxi School of Medicine, Jiangnan University, Wuxi, China; 5 Division of Rheumatology, Department of Medicine Solna, Karolinska Institutet, Karolinska University Hospital, Stockholm, Sweden; 6 Department of Medical Biochemistry and Biophysics, Karolinska Institutet, Stockholm, Sweden; 7 Division of Neurogeriatrics, Department of Neurobiology, Care Sciences and Society, Karolinska Institutet, Stockholm, Sweden; 8 Science for Life Laboratory, Department of Gene Technology, KTH Royal Institute of Technology, Stockholm, Sweden; 9 Institut de Génomique Fonctionnelle, Université de Montpellier, CNRS, INSERM, Montpellier, France; 10 Jinfeng Laboratory, Chongqing, China; 11 Department of Physiology and Pharmacology, Center for Molecular Medicine, Karolinska Institutet, Stockholm, Sweden

**Keywords:** Microglia, Oligodendrocyte, Multiple sclerosis

Correction to: *Nature Neuroscience* 10.1038/s41593-025-02161-4, published online 2 January 2026.

In the version of the article initially published, in Fig. 7a, “*Tgfbr2*^fl/fl^” should have read “*Tgfb1*^fl/fl^” and in Fig. 7b, d, e and f, all instances of “*Tgfbr1*^fl/fl^” should have read “*Tgfb1*^fl/fl^”. These corrections have been made to the HTML and PDF versions of the article.

